# Mobilisation Mechanism of Pathogenicity Islands by Endogenous Phages in *Staphylococcus aureus* clinical strains

**DOI:** 10.1038/s41598-018-34918-2

**Published:** 2018-11-13

**Authors:** Mercedes Cervera-Alamar, Katerina Guzmán-Markevitch, Miglė Žiemytė, Leticia Ortí, Patricia Bernabé-Quispe, Antonio Pineda-Lucena, Javier Pemán, María Ángeles Tormo-Mas

**Affiliations:** 1 Severe Infection Group, Health Research Institute Hospital La Fe, Valencia, Spain; 2 Drug Discovery Unit, Health Research Institute Hospital La Fe, Valencia, Spain; 3 Joint Research Unit in Clinical Metabolomics, Príncipe Felipe Research Center/Health Research Institute Hospital La Fe, Valencia, Spain; 4 Microbiology Department, Polytechnic University Hospital La Fe, Valencia, Spain

## Abstract

*Staphylococcus aureus* pathogenicity islands (SaPIs) are a type of mobile genetic element that play a significant role in the pathogenesis and virulence of this microorganism. SaPIs are integrated in the chromosome under the control of the master repressor Stl, but they can be horizontally transferred at a high frequency due to certain bacteriophages. Thus, a phage protein can bind to the SaPI Stl and induce the SaPI cycle, spreading the SaPI virulence factors to other bacterial populations. We report the dissemination mechanism of SaPIs mediated by endogenous prophages in *S. aureus* clinical strains. We reveal the induction of SaPIs by a co-resident prophage in seven clinically relevant strains, and we further study this mechanism in MW2, a community-acquired methicillin-resistant *S. aureus* strain that contains two bacteriophages (ɸSa2mw and ɸSa3mw) and one SaPI (SaPImw2) encoding for three enterotoxins (*sec, sel and ear*). ɸSa2mw was identified as responsible for SaPImw2 induction, and the specific phage derepressor protein DUF3113 was determined. The Stl-DUF3113 protein interaction was demonstrated, along with the existence of variants of this protein in *S. aureus* phages with different abilities to induce SaPI. Both Stl and DUF3113 are present in other *Staphylococcus* species, which indicates that this is a generalised mechanism.

## Introduction

*Staphylococcus aureus* is a widespread pathogen that colonises the skin and mucosa of healthy adults. *S. aureus* is found in both hospital and community environments, causing a variety of infectious diseases from minor skin infections to serious conditions such as osteomyelitis or endocarditis^1,2^. The high pathogenicity of this microorganism is due to the broad spectrum of virulence factors present in its genome. Remarkably, many of these virulence factors such as toxins, adhesins or invasins are part of its mobile genetic elements (MGE)^3,4^.

The acquisition of most MGEs is produced by horizontal transfer, *Staphylococcus aureus* pathogenicity islands (SaPIs) and the bacteriophages are two closely related MGEs that have been identified in *S. aureus* and whose mobilisation mechanisms have been studied^4,5^. SaPIs contain virulence genes that can be disseminated amongst bacteria populations by means of certain phages^6,7^. SaPIs act as molecular parasites, using different mechanisms to interfere with the normal biology of phages, to encapsidate in phage particles and to promote their own spread^8–12^.

SaPIs, as bacteriophages, can be packaged using two different mechanisms: *pac* and *cos*^13^. They can be packaged in full-sized procapsids by *pac* phages or packaged in DNA units delimited by *cos* sites in *cos* phages. Additionally, *pac* SaPIs can interfere with *pac* phages via several proteins such as TerS or CpmAB to reduce the capsid size and exclusively pack the island genome^14–16^. Recently, SaPIs that do not encode TerS or CpmAB homologues have been identified, they are *cos* SaPIs and they can use the packaging mechanisms of *pac* and *cos* phages^17^. These *cos* SaPIs encode for Ccm, which is responsible for small capsid formation and interferes with the packaging mechanism of *cos* phages^18^.

**Figure 1:**
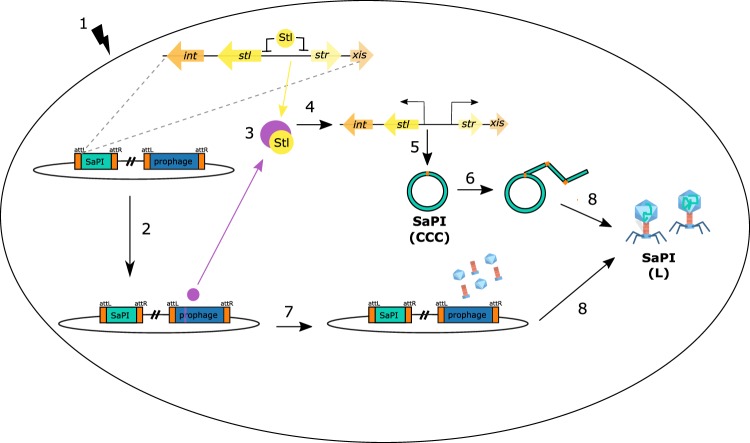
SaPI cycle after prophage induction. (1) SOS response after mitomycin C addition. (2) The prophage is induced and start the gene transcription, while the SaPI resides passively in the genome under the control of the global repressor Stl. (3) The interaction between the Stl and a specific phage derepressor protein induces the SaPI cycle. (4) The SaPI gene transcription starts. (5) SaPI is excised from the chromosome in covalently closed circular (CCC) state and (6) starts the replication. (7) The phage synthetises the procapsids and tails. (8) SaPI L indicates linear monomers released from the phage heads if the prophage is able to mobilise the SaPI.

In general, SaPIs are repressed and integrated in the host chromosome. The normal repressed state of SaPIs is due to the master regulator Stl^19^. Stl acts as a repressor, binding to a region between two divergent promoters and inhibiting the transcription of SaPI genes^20,21^. The SaPI life cycle starts by the infection of a helper phage or by activation of an endogenous helper prophage by the SOS response. This response can be caused by oxidative stress, exposure to UV-irradiation or antibiotic treatment^22–24^. Once the prophage is activated or the cell is infected by the phage, a bacteriophage protein binds to Stl, triggering the release of the Stl repressor from the SaPI DNA. Disruption of the Stl-SaPI DNA complex allows the expression of SaPI proteins and the initiation of the excision-replication-packaging (ERP) cycle of the SaPI^25^ (Fig. 1).

The interaction between Stl and the phage derepressor protein is the first step of the SaPI cycle. In the different SaPIs, Stl sequences are not conserved. On the other hand, derepressor proteins are specific for each interaction. Therefore, bacteriophages have different capacities to induce SaPIs according to whether they present derepressor proteins which are able to bind to specific Stls. All phage derepressor proteins studied so far are known as moonlighting proteins because they have more than one function, apart from their role in SaPI induction, a second function is supposed to be beneficial for the phage. The functions of four bacteriophage proteins have already been described. Dimeric and trimeric dUTPases of various phages are derepressor proteins of SaPIbov1 and SaPIbov5. Sri protein, which binds to DnaI and inhibits host cell replication, is the derepressor protein of SaPI1. SaPIbov2 was shown to be induced by ORF15 of the 80α phage, but its function still remains unknown^25–28^ and the single-strand annealing protein (SSAP) family, involved in homologous recombination, induces SaPI2^29^. These four phage derepressors are able to induce only a few SaPIs. Furthermore, it has been shown that Stl repressors may have different domains to interact with unrelated phage proteins but perform the same conserved function^29,30^. Therefore, there are still many uncertainties about SaPI induction, and the identification of new derepressor proteins is key to understanding this induction mechanism and the subsequent dissemination to other bacteria.

SaPI induction by helper phages has been studied in phi11, phi12 and 80α lysogens, but there has been no demonstration of SaPI induction by endogenous phages in clinical strains. In this work, we demonstrate the ability of certain quiescent bacteriophages to induce resident SaPIs. We studied the SaPI induction process in 14 *S. aureus* clinical strains and focused on SaPImw2 induction in the MW2 strain. MW2 is a community-acquired methicillin-resistant *S. aureus* (MRSA) strain that presents 19 unique toxins^31,32^, some of them encoded in the SaPImw2 pathogenicity island. We demonstrated that the ɸSa2mw phage is able to induce SaPImw2, and we identified the phage derepression protein responsible for the induction and horizontal transference of the SaPImw2 genome. Finally, derepressor homologous proteins were identified in various phages. All of the proteins showed different abilities to interact with Stl and, consequently, different capacity to induce SaPImw2.

## Results

### SaPI induction by endogenous phages in clinical strains

SaPI induction by prophages has been extensively studied in laboratory strains, mainly in lysogens from different phages in the non-lysogenic strains RN4220 and RN450. To confirm whether this mechanism occurs naturally, a total of 14 *S. aureus* clinical strains were treated with mitomycin C to activate the SOS response and induce endogenous phages to check whether these phages are able to activate the resident SaPIs (Fig. 1). The clinical *S. aureus* strains MRSA252, E-MRSA16, Mu50, TW20, MN8, USA300, COL, N315, MW2, A, B, C, D and E^33^, were selected because of their broad characterisation and because their genomes have already been sequenced. All of these strains contain SaPIs and bacteriophages integrated in the chromosome (Table 1).

**Table 1:** Description of the clinical strains used in the study.

Strain	SaPI inducible by endogenous phage	SaPI (packaging type)	SaPI Integrase	Prophage (packaging type)	Phage derepressor protein
E-MRSA16	no	SaPI4	I	φSa2, φSa3	—
MRSA252	no	SaPI4	I	φSa2, φSa3	—
Mu50	no	SaPIm4, SaPIm1	III, V	φSa3	—
TW20	no	SaPI1	IV	φSa1, φSa3	—
MN8	no	SaPI2	V	φSa1, φSa2, φSa3 φSa5, φSa6	—
USA300	yes	SaPI5 (*cos*)	IV	φSa2usa (*pac*), φSa3usa (*cos*)	unknown
COL	yes	SaPI3 (*pac*)	IV	φCOL (*cos*)	unknown
N315	yes	SaPIn1(*pac*)	V	phiN315 (φSa3) (*cos*)	Redβ
MW2	yes	SaPImw2 (*cos*)	III	φSa2mw (*cos*), φSa3mw (*pac*)	DUF3113
A	yes	SaPIa (*pac*)	I	φSa3 (*cos*)	Sri
B	no	SaPIb	I	φSa2, φSa3	—
C	yes	SaPIc (*cos*)	III	φSa2, φSa6 (*cos*)	DUF3113
D	no	SaPId	V	φSa1, φSa3, φSa6	—
E	yes	SaPIe (*pac*)	I	φSa3 (*cos*)	unknown

**Figure 2:**
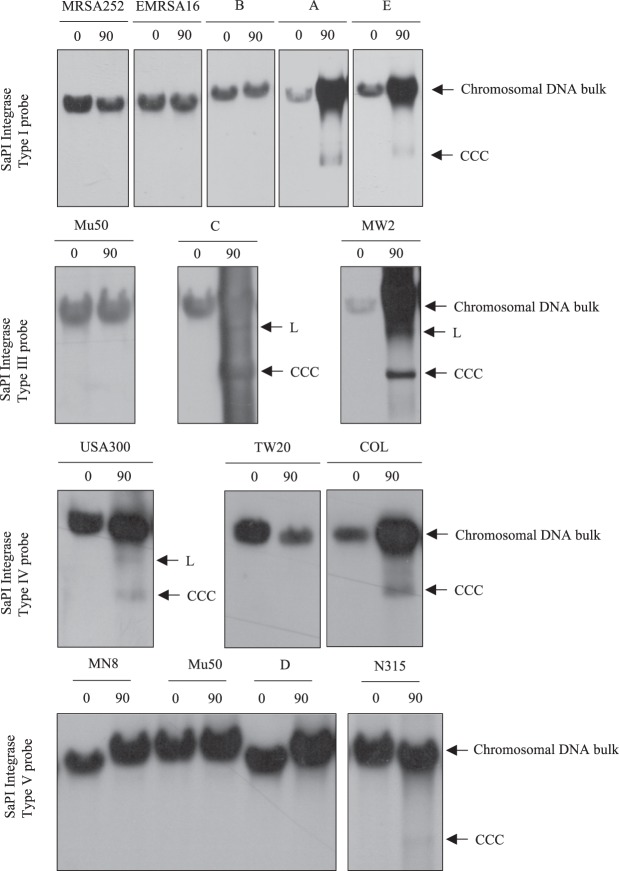
SaPI induction of *S. aureus* clinical strains by endogenous phages. In total, 14 strains were treated with mitomycin C (2 µg/mL) to activate the SOS response and induce the resident prophages. Samples were isolated at 0 and 90 minutes, separated in agarose gel and blotted with the SaPI integrase-specific probe. The upper band corresponds to chromosomal bulk DNA including SaPI, and the lower bands correspond to covalently closed circular SaPI (CCC) excised from the genome or SaPI monomers released from the phage capsid (L) if the helper phage mediated induction and packaging.

SaPI induction was tested by Southern blot, as indicated in the methods section. The blots showed that 7 strains contain prophages able to induce the resident SaPIs (Fig. 2). In MW2, C and USA300 strains the hybridization signal of the specific probe for each SaPI was observed in the bulk DNA as well as in a band that corresponds to SaPI linear monomers (L) released from the small phage capsids, as it was observed in previous works, in presence of a phage able to induce and encapsidate the SaPI^25,34^. Instead, a second band in A, E, N315 and COL strains, at the bottom of the gel (Fig. 2), is the covalently closed circular molecules (CCC) which correspond to the SaPI induced but not packaged^25,34,35^.

To explain the absence of an L SaPI band in A, E, N315 and COL strains, corresponding with SaPIs that have been packaged, the SaPI and prophage sequences of the clinical strains were analysed to identify whether they are *cos* or *pac* types (Table 1). *pac* SaPIs contain a group of genes related to phage packaging and interference, known as Operon I, and a small terminase that recognises the unique *pac* site. In contrast, *cos* SaPIs present a highly conserved phage *cos* site and a different packaging and interference module (Operon 1 like), and lacks the small terminase. The differences in migration and the assignment in two types (CCC or L) of the lower band among the analysed strains are consistent with the presence of *cos* and *pac* SaPIs and phages. MW2, C and USA300 encode *cos* SaPI and *cos* phages with similar *cos* sites, so the SaPIs can be induced and mobilised by their endogenous phages. A, E, N315 and COL contain *pac* SaPIs and *cos* phages, so there might be induction but not packaging in these strains.

After induction, SaPI is excised from the chromosome and circularised. To clearly confirm that SaPI induction has occurred, SaPI excision from the chromosome was confirmed by PCR using specific primers recognising the flanking region of each SaPI integrase type (Supplementary Fig S1). After treatment with mitomycin C, all of the strains that produced a band that migrated at a position suggesting SaPI induction by Southern blot analysis could be confirmed as the excised product by PCR. Additionally, SaPI circularisation was tested by PCR using a pair of primers set divergently at both termini of each SaPI. Only the strains positive for SaPI induction were tested, and all showed a band indicating SaPI circularisation (Supplementary Fig. S2).

These results confirm that the SaPIs present in the strains A, E, C, MW2, USA300, COL and N315 are able to excise from the chromosome and circularisation after induction.

Analysis of the global repressor *stl* sequences of the 7 induced SaPIs revealed that the *stl* gene is highly divergent among different SaPIs^19^ (Supplementary Fig. S3). Some SaPIs contain identical *stls* such as USA300 and COL or MW2 and C. The SaPI from strain A contains an *stl*, which has already been described, and the derepressor was identified as a Sri protein^25^. A Redβ protein was recently identified as the derepressor of SaPI-N315^29^. In contrast, the other Stl repressor have not yet been studied, and the phage proteins responsible for interaction with them remain unknown (Table 1).

In this study, we paid special attention to the SaPI mobilisation mechanism of these clinical strains. We focused on identifying the phage protein responsible for induction of the SaPIs present in the C and MW2 strains, which encode for the same *stl*. MW2 is a community-acquired methicillin-resistant *S. aureus* strain that encodes multiple virulence factors, some of them in SaPImw2. Consequently, it is important to understand how this SaPI is mobilised and how its virulence factors are spread.

### ɸSa2mw from MW2 induces SaPImw2 replication and transfer

To follow SaPImw2 replication and mobilisation, a chloramphenicol (Cm) marker was introduced in the SaPI genome, which resulted in strain GTM750 (Supplementary Table S1). The chloramphenicol acetyl transferase (*cat*) gene was inserted, replacing enterotoxin type C (*sec4*), an accessory gene of the pathogenicity island. SaPImw2 belongs to a SaPI subfamily in which the classical SaPI operon I is not present and contains a conserved phage *cos* site sequence (cggcgggggc)^17,18,35^. As SaPImw2 is a *cos* SaPI, in the replacement, the SaPI genome size must not be affected, because the SaPI size genome is a prerequisite for high-frequency SaPI transfer by *cos* phages^18^.

ɸSa2mw and ɸSa3mw phages were identified in the human clinical strain MW2 genome. ɸSa2mw is a *cos* phage that presents the same *cos* site sequence as that of SaPImw2. For this reason, we hypothesised that ɸSa2mw could be responsible for SaPImw2 mobilisation. To examine this idea, we generated ɸSa2mw lysogen in the non-lysogenic strain RN4220, which yielded strain GTM752. In addition, SaPImw2 *ent*C*::cat* was transduced into the GTM752 strain, which generated GTM753.

**Figure 3:**
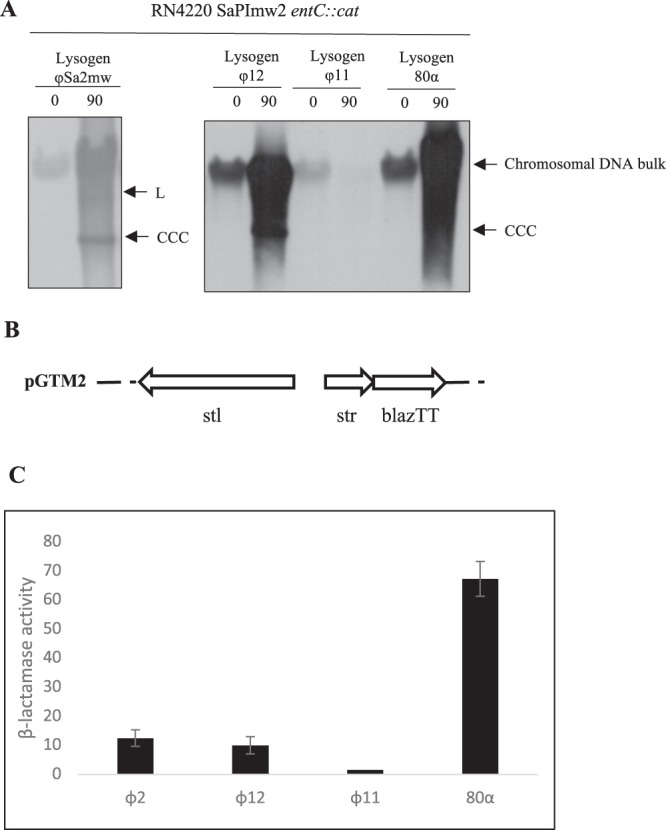
(**A**) Southern blot of φSa2mw, φ12, φ11 and 80α lysogens in SaPImw2-positive strains (blotted with SaPI integrase type III probe) (**B**). Diagram of the derivative pCN41 (pGTM2), which contains the regulatory region of SaPImw2 fused to blazTT. (**C**) The lysogen of phages φSa2mw, φ12, φ11 and 80α containing pGTM2 were assayed for β-lactamase activity after 2 h of induction with mitomycin C (2 µM). Data are result of three independent experiments.

SaPImw2 induction of GTM753 was tested by Southern blot (Fig. 3A), and we demonstrated that ɸSa2mw has the ability to mobilise SaPImw2 *ent*C*::cat* to a recipient strain RN4220 from GTM753 (Table 2). On the other hand, SaPIs interfere with the reproduction of their helper phage, blocking plaque formation and reducing plaque number^36,37^. The phage titre was reduced by more than one order of magnitude in presence of SaPImw2 *ent*C*::cat* (GTM753) with regard to RN4220 ɸSa2mw (GTM752), which confirmed the interference. The relationship between SaPI and the bacteriophage is a parasitism relationship, in which SaPI benefits but the phage is impaired (Table 2). All these results indicate that ɸSa2mw is responsible for the SaPImw2 induction and packaging in small capsids.

**Table 2:** SaPI and phage titres.

	Strain	Phage	SaPI	Plasmid	phage titer^Т^ (pfu/ml)	SaPI titre* (cfu/ml)
	MW2	φSa2mw, φSa3mw	SaPImw2 *ent*C*::cat*		4.82E + 05	5.50E + 07
GTM752	RN4220	φSa2mw			2.86E + 03	
GTM753	RN4220	φSa2mw	SaPImw2 *ent*C*::cat*		9.32E + 01	4.69E + 04
GTM758	RN4220	φSa2mwΔDUF3113			4.38E + 03	
GTM757	RN4220	φSa2mwΔDUF3113	SaPImw2 *ent*C*::cat*		2.25E + 04	0.00E + 00
GTM759	RN4220	φSa2mwΔDUF3113	SaPImw2 *ent*C*::cat*	pCN51 DUF3113	7.00E + 01	2.04E + 05
	RN451	φ11			2.77E + 09	
GTM785	RN451	φ11	SaPImw2 *ent*C*::cat*		9.62E + 07	7.00E + 00
GTM806	RN451	φ11ΔDUF3113	SaPImw2 *ent*C*::cat*		5.92E + 08	0.00E + 00
	RN10359	80α			1.79E + 10	
GTM784	RN10359	80α	SaPImw2 *ent*C*::cat*		6.87E + 08	3.21E + 03
	RN4220	φ12			2.05E + 05	
GTM786	RN4220	φ12	SaPImw2 *ent*C*::cat*		4.00E + 03	1.71E + 05
GTM807	RN4220	φ12ΔDUF3113	SaPImw2 *ent*C*::cat*		9.23E + 04	0.00E + 00

^**Т**^Phage titre using RN4220 as an indicator.

*Transfer of SaPImw2 using RN4220 as a recipient.

The results are the means of three independent experiments, and the variation is within 5%.

Previous studies have demonstrated that the interaction of a phage protein with the Stl repressor interrupts the binding of Stl to the intergenic region of the SaPI regulation module, which causes initiation of the SaPI cycle^25^. To demonstrate this mechanism for MW2, we constructed the plasmid pGTM2 (Supplementary Table S2), a derivative pCN41 with the reporter gene β-lactamase fused with the intergenic regulatory region of SaPImw2, including the *stl* gene (Fig. 3B). pGTM2 was introduced into the lysogenic strain GTM752 of ɸSa2mw, which created GTM754. The strain was then induced with mitomycin C, and the expression of the β-lactamase reporter was measured as indicated in the methods section. As is shown in Fig. 3C, expression of the β-lactamase reporter gene appeared when phage ɸSa2mw was induced, so we concluded that ɸSa2mw activates transcription by specifically disrupting the Stl–DNA complex.

### DUF3113 is responsible for derepression of SaPImw2

**Figure 4:**
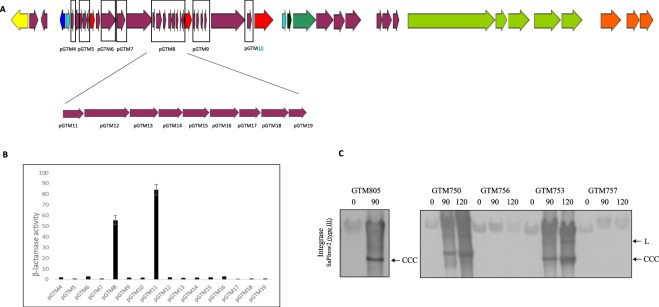
(**A**) Diagram of the ɸSa2mw genome with the cloned regions marked. (**B**) β-lactamase activity assay of the derivative pGTM3 (pCU1_P*cad*) with different regions of φSa2mw in GTM754 (RN4220 φSa2mw pGTM2) strains. (**C**) Southern blot of GTM805 containing SaPImw2 and complemented with a plasmid overexpressing DUF313_ɸSa2mw_. One millilitre of the culture was collected after 2 h of induction with 2 μM of CdCl_2_. The lower band corresponds to CCC because there was no helper phage present. Southern blot was performed with the wild-type strains (GTM750 and GTM753) and the DUF3113 mutant deletion in MW2 (GTM756) and GTM753 (GTM757). Both Southern blots were hybridised with a SaPI integrase type III probe.

To identify which protein of ɸSa2mw could be responsible for the interaction with Stl and, therefore, for SaPImw2 induction, a total of 7 regions of ɸSa2mw were cloned into the plasmid pGTM3 (pCU1_P*cad*) (Fig. 4A). Due to the previously described inducers are encoded in the replication module, the 7 selected regions correspond to this region. The cloning resulted in plasmids pGTM4 to pGTM10, which were introduced into GTM755 (RN4220 pGTM2) and induced with CdCl_2_ (2 µM). As shown in Fig. 4B, pGTM8 was responsible for the activity of β-lactamase. The region of pGTM8 is composed of 9 open reading frames (ORFs), from MW1424 to MW1416. All of these ORFs were cloned individually into pGTM3 (pCU1_P*cad*), generating 9 new plasmids (from pGTM11 to pGTM19), and were transformed into GTM755 (RN4220 pGTM2). In this case, pGTM11 showed β-lactamase activity and corresponded to ORF MW1424. This ORF encodes for a hypothetical protein, DUF3113 (UniProtKB code A0A0H3JWD1_STAAW), which belongs to the DUF3113 superfamily.

Previous studies have indicated that SaPIs protect bacteria from exogenous phage-mediated infection, as long as this exogenous phage is able to induce the SaPI cycle^37^. Furthermore, it has also been noted that phages with mutations in the derepressor gene are able to form plaques in strains that contain an inducible SaPI^29,36,37^. We demonstrate in this study that SaPImw2 blocks plaque formation by ɸSa2mw. However, after several attempts to infect a SaPImw2-positive strain (GTM751) with ɸSa2mw lysate, two plaques were obtained. The sequence of the DUF3113 derepressor gene from the phage genomes obtained from the plaques showed mutations in the ribosome-binding site and one mutation in the start codon (atg to atc; M1I), which suggests that this gene is responsible for SaPImw2 induction (Supplementary Fig. S4).

Overexpression of DUF3113 by cloning into a vector pCN51, producing pGTM20, and transforming into the RN4220 SaPImw2 *ent*C*::cat*, generating GTM805 strain, confirmed that DUF3113 is the unique protein responsible for SaPImw2 induction (Fig. 4C). Moreover, an inframe deletion using allelic exchange with the pMAD vector in this gene was carried out in GTM750 (MW2 SaPImw2 *ent*C*::cat*) and in GTM753 (RN4220 ɸSa2mw SaPImw2 *ent*C*::cat*), which generated GTM756 and GTM757, respectively. To confirm our previous results, SaPImw2 mobilisation was tested by Southern blot. No SaPI specific band was detected when DUF3113 was not present (Fig. 4C).

Finally, to confirm that DUF3113 is responsible for SaPImw2 mobilisation, SaPI and phage titres were studied by induction with mitomycin C of the ɸSa2mw lysogen wt and DUF3113 mutant strain and in the absence and presence of SaPImw2 *ent*C*::cat* (Table 2). Phage titres indicate that the phage mutant seems to be as viable as the wild type. Therefore, DUF3113 gene is not essential for the phage in laboratory conditions. On the other hand, the mutant phage was not able to transduce SaPImw2*ent*C*::cat*, but when DUF3113 was overexpressed (pGTM20) in the mutant strain (GTM759), the SaPI titer was fully restored.

### DUF3113 interacts with SaPImw2 Stl

**Figure 5:**
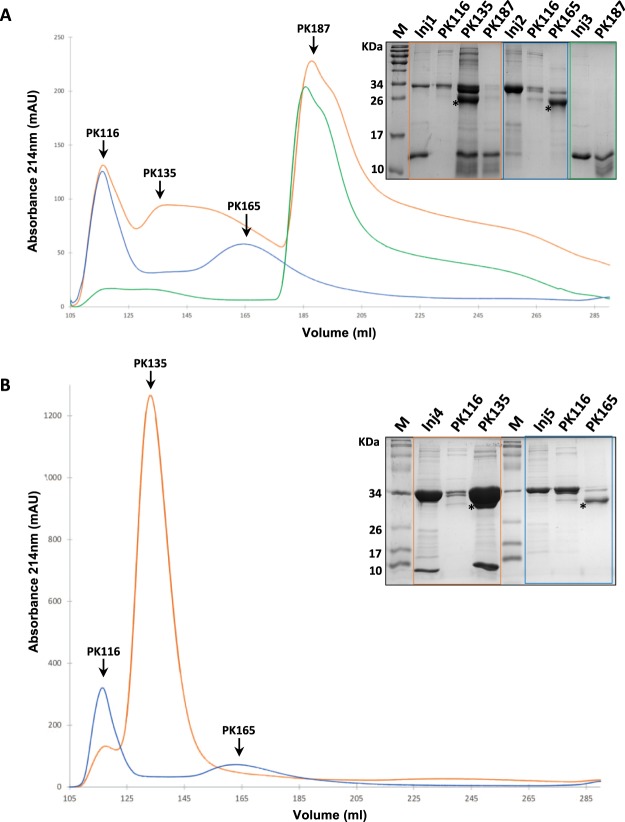
(**A**) Size-exclusion chromatography assays. Blue: Free His6-Stl_SaPImw2_ chromatogram (PK116 and PK165)_._ Green: His6-DUF3113_ɸSa2mw_ chromatogram (PK187). Orange: Chromatogram corresponding to the interaction between His6-Stl_SaPImw2_ and His6-DUF3113_ɸSa2mw_. As confirmed by SDS-PAGE (top insert), PK116 belonged to His6-Stl_SaPImw2_, PK187 to His6-DUF3113_ɸSa2mw_ and PK135 to the complex between the two proteins. (**B**) Size exclusion chromatography of the pull-down assays. Orange: Chromatogram corresponding to the pull-down of His6-Stl_SaPImw2_ with untagged DUF3113_ɸSa2mw_, in which one peak was observed for PK135, corresponding to the interaction of both proteins. Blue: Chromatogram of the pull-down of His6-Stl_SaPImw2_ with untagged DUF3113_ɸSa2c_. Two peaks, PK116 and PK165, corresponding to free His6-Stl_SaPImw2 _were observed. All peaks were analysed by SDS-PAGE. Inj1: injection of His6-Stl_SaPImw2_ (32,5 KDa) and His6-DUF3113_ɸSa2mw_ (10.6 KDa); Inj2: injection of His6-Stl_SaPImw2_; Inj3: injection of His6-DUF3113_ɸSa2mw;_ Inj4: injection corresponding to the pull-down of His6-Stl_SaPImw2_ - untagged DUF3113_ɸSa2mw_ (7.1 KDa); and Inj5: injection corresponding to the of pull-down His6-Stl_SaPImw2_-untagged DUF3113_ɸSa2c_ (7.1 KDa). A degradation product from protein His6-Stl_SaPImw2_ was observed in PK165 (asterisk).

SaPI induction occurs through direct protein interaction between the phage derepressor protein and the SaPI repressor Stl^25,38^. To test the *in vitro* interaction between DUF3113_ɸSa2mw_ and Stl_SaPImw2,_ a size-exclusion chromatography assay was performed, and all the relevant peak fractions were analysed by SDS-PAGE. His6-tagged Stl_SaPImw2_ and His6-tagged DUF3113_ɸSa2mw_ were expressed (plasmids pGTM22 and pGTM23) and purified. Figure 5A shows three chromatograms of His6-Stl_SaPImw2_, His6-DUF3113_ɸSa2mw_ and the complex His6-Stl_SaPImw2_-His6-DUF3113_ɸSa2mw_. The His6-Stl_SaPImw2_ chromatogram (blue) showed two peaks (PK116 and PK165), and the His6-DUF3113_ɸSa2mw_ chromatogram (green) showed one peak (PK187). However, the complex His6-Stl_SaPImw2_ - His6-DUF3113_ɸSa2mw_ (orange) eluted in 3 peaks: PK116, PK135, PK187. PK116 and PK187 corresponded to specific elution volumes from free His6-Stl_SaPImw2_ and His6-DUF3113_ɸSa2mw_, respectively. However, the new peak, PK135, was associated with the protein complex, as was confirmed by SDS-PAGE.

In addition, to confirm the Stl-DUF3113 interaction, pull-down assays were performed. For this, His6-tagged Stl_SaPImw2_ and untagged DUF3113_ɸSa2mw_ were expressed (plasmids pGTM22 and pGTM24), and His6-tagged Stl_SaPImw2_ was bound to a metal affinity resin and incubated with untagged DUF3113_ɸSa2mw_. The co-elution of both proteins was confirmed by SDS-PAGE (Supplementary Fig. S5) and by size-exclusion chromatography (Fig. 5B). Again, a peak at 135 mL was detected, corresponding to the interaction of both proteins, as confirmed by SDS-PAGE. The negative control, untagged DUF3113_ɸSa2mw_, was not retained by the resin (data not shown). Furthermore, to check the specificity of Stl_SaPImw2_-DUF3113_ɸSa2mw_, the Stl of SaPIbov1, which is not induced by DUF3113_ɸSa2mw_, was used as a control (His6-Stl_SaPIbov1_). This protein construct was also expressed and incubated with untagged DUF3113_ɸSa2mw_; however, in this case, no interaction was detected (Supplementary Fig S5). Finally, the interaction between His6-Stl_SaPImw2_ and DUF3113_ɸSa2c_, which does not derepress SaPImw2 (see below), was evaluated and, as expected, they did not co-elute (Fig. S5). The lack of interaction between these two proteins was also confirmed by size-exclusion chromatography (Fig. 5B). Here, only two peaks (PK116 and PK165) were detected, corresponding to His6-Stl_SaPImw2_.

All these results indicate that DUF3113_ɸSa2mw_ binds directly and specifically to Stl_SaPImw2._

### Diverse DUF3113 phage proteins display different capacities to induce SaPImw2

Because there are significant differences in the bacteriophage capacities to induce SaPIs^8^, the ability of different phages such as 80α, ɸ11 and ɸ12 to induce SaPImw2 was tested by Southern blot (Fig. 3A). ɸ11 cannot induce SaPImw2, whereas 80α and ɸ12 are able to induce SaPImw2. ɸ12 is a *cos* phage like ɸSa2mw and contains the same *cos* sequence. In the same way as that shown for ɸSa2mw, SaPImw2 induction by ɸ12 showed a lower band corresponding to a SaPI monomer packaged in small capsids. Most likely, this was due to the presence of the *ccm* gene in SaPImw2, which is responsible for conducting packaging in small capsids. On the other hand, Ccm protein is responsible for *cos* phage interference^18^. In contrast, 80α is a *pac* phage, and there was no lower band after induction, probably because SaPImw2 does not encode for CpmA and CpmB, which are responsible for the formation of smaller capsids than those normally generated by the *pac* phages.

The transducing titres of SaPImw2 *ent*C*::cat* (SaPI titres) showed that 80α and ɸ12 mobilised this SaPI successfully. As expected, ɸ11 was unable to transfer SaPImw2 (Table 2). To study the capacity of the phages to bind to Stl_SaPImw2_ and increase β-lactamase expression, the plasmid pGTM2 was introduced in 80α, ɸ11 and ɸ12 lysogens (Fig. 3C). All tested phages showed different abilities to derepress the regulatory region and induce SaPImw2.

**Figure 6:**
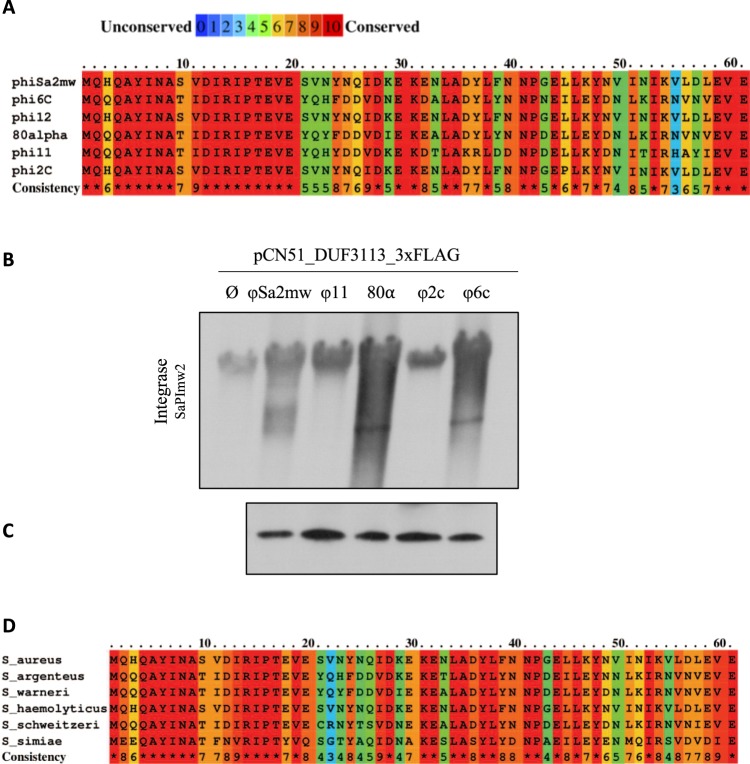
(**A**) Sequence alignment of DUF3113 homologues in *S. aureus* phages using PRALINE^45^. The scoring scheme assigns 0 as the least-conserved alignment position, with values up to 10 (asterisk) for the most-conserved alignment position (**B**). Southern blot of SaPImw2 excision after induction of 3xFLAG-tagged homologues to the DUF3113 protein from different phages cloned into pCN51 after 2 h of CdCl_2_ treatment. (**C**) Western blot of 3xFLAG-tagged DUF3113 homologues cloned into pGTM3 (pCU1_ P*cad*) with the same RBS (**D**). Sequence alignment of DUF3113 homologues of different staphylococcal species.

An *in silico* approach was carried out to look for DUF3113_ɸSa2mw_ homologues in these phages and check whether the allelic variants of DUF3113 were also able to induce SaPImw2. The phage ɸ12 contains a protein with 100% identity with DUF3113_ɸSa2mw_. Phages 80α and ɸ11 encode DUF3113_ɸSa2mw_ homologues in ORF25 and ORF19, respectively, both with 61% amino acid identity. As mentioned earlier, strain C, with a resident SaPI also mobilised by an endogenous bacteriophage (Fig. 2), carries two prophages in its genome. These phages have been designated ɸ2c and ɸ6c in this study. Both phages also present DUF3113_ɸSa2mw_ homologues with 98% and 58% amino acid identity, respectively (Fig. 6A).

To determine whether the different DUF3113 proteins are able to induce SaPImw2, DUF3113 genes from phages 80α, ɸ11, ɸ2c and ɸ6c were tagged with 3xflag, cloned into the plasmid pCN51 and introduced into the RN4220 SaPImw2*ent*C*::cat* (GTM751) strain. SaPImw2 induction was tested by Southern blot after treatment with CdCl_2_ (Fig. 6B). DUF3113 from phages 80α and ɸ6c induced SaPImw2, while the homologous protein from ɸ11 and ɸ2c could not induce it.

As in the previous experiment using lysogens, the β-lactamase expression was measured after cloning DUF3113 homologues genes from different phages into pGTM3 (pCU1_P*cad*) tagged with 3xflag and then transformed into GTM755 (RN4220 pGTM2). The β-lactamase activity varied between the tested strains, which supported the Southern blot results (Fig. 6B). However, after Western blotting was performed, differences in the levels of protein expression were observed. These differences could be due to either the expression level of the derepressor phage proteins or to differences in the amino acid sequences of these proteins. To this end, we cloned the homologous proteins tagged with 3xflag with the same ribosome binding site (RBS of DUF3113) into pCN51 and pCN41 plasmids, which were then transformed into GTM751 and GTM755 (RN4220 pGTM2), respectively. Southern blot showed the same differences in SaPImw2 induction as those seen in Fig. 6B when the proteins were equally expressed (Fig. 6C). Different allelic variants differ in their capability to derepress SaPImw2 due to differences in the ability to bind to Stl_SaPImw2._

To determine whether DUF3113 is also present in another *Staphylococcus* spp, an *in silico* study was performed. We found DUF3113 protein in phages from *Staphylococcus argenteus, Staphylococcus warneri*, *Staphylococcus haemolyticus*, *Staphylococcus schweitzeri* and *Staphylococcus simiae* (Fig. 6D). In addition, we discovered an *S. argenteus* clinical strain, BN75, which contains a SaPI that encodes for a Stl similar to Stl_SaPImw2_ (99% of identity) and a phage with a DUF3113 protein similar to DUF3113_ɸSa2mw_ (61% identity). In the same way, we located a SaPI that encodes for a Stl and a phage with a DUF3113 in the *S. aureus* strain TCH70 showing 100% identity to present in MW2. These results demonstrate that the mechanism described is general in *Staphylococcus* spp.

## Discussion

SaPIs encode for virulence factors such as superantigens, epidermolytic toxins, biofilm formation genes, and iron transport or antibiotic-resistance genes (penicillin, fosfomycin and fusidic acid). Therefore, these MGE have a high clinical relevance^34^. For this reason, it is very important to know how these SaPIs are mobilised and, consequently, how they spread their virulence factors among bacteria of the same species and even among bacteria of different genera. In this paper, we characterised the mobilisation mechanism of SaPImw2 carrying genes for enterotoxin *sec*, *sel* and *ear*. The last of the listed genes is linked to ampicillin resistance.

The Stl sequences of the SaPIs are not conserved among different families, and, therefore, each SaPI family is induced by different phage proteins^25^. There are many SaPIs with unknown inducers. It has recently been demonstrated that the Stl sequences of certain SaPIs have acquired multiple domains to bind to different phage inductor proteins^29^. On the other hand, SaPIs and bacteriophages are in continuous co-evolution. Subsequently, much remains to be understood about the SaPI induction mechanism.

Prophages and SaPIs are abundant in the *S. aureus* genome. Most strains contain more than one prophage and at least one SaPI^39^. Based on the fact that SaPI interference mechanisms are counterselective for the phages and that the phages capable of relieving Stl repression are not able to form plaques on a SaPI-containing strain^25^, endogenous phages able to induce co-resident SaPIs are considered unlikely to be found. However, this study showed that SaPI induction by endogenous phages naturally occurs in human clinical *S. aureus* isolates, as we demonstrated for the MW2, USA300, COL, N315, A, C and E strains.

There are some possible explanations for this finding. SaPIs have different interference mechanisms that negatively affect the phage packaging machinery. On the one hand, *pac* SaPIs encode for the genes *cpm*A*, cpm*B*, ppi, pti*A*, pti*B *and pti*M, which are responsible for interference with *pac* phage packaging. On the other hand, *cos* SaPIs, which encode the *ccm* gene, cause severe interference in *cos* phage packaging^18^. However, there are no described interference mechanisms between *pac* SaPIs and *cos* phages. Therefore, in strains COL, N315, A and E, in which the SaPIs are *pac* and their co-resident phages are *cos*, bacteriophages may have been acquired by horizontal transfer regardless of the SaPI presence, since they were not affected by interference mechanisms.

In contrast, MW2, as described, contains the *cos* SaPI SaPImw2, which is induced and packaged by the *cos* phage ɸSa2mw. In this case, we think that SaPImw2 may have been acquired after the phage.

Finally, strain C has two *cos* prophages and one *cos* SaPI. We describe that the *cos* SaPIc is induced by *cos* ɸ6c, but probably, this phage is not responsible for packaging because the *cos* site and its flanking region, which is important for mobilisation and packaging, are completely different between SaPIc and ɸ6c. However, ɸ2c does present a *cos* site and its flanking region with 89% identity with SaPIc, which suggests that this phage is probably able to mobilise this SaPI. Therefore, the assumption is that ɸ6c is the responsible for the induction, while ɸ2c is mainly involved in packaging. Phage major capsid protein (CP) is the target for Ccm SaPI protein interference. These two proteins present in the C-terminal have high structural similarity^18^. In our case, Ccm SaPIc presents a high structural similarity with CP_ɸ2c_ and no similarity with CP_ɸ6c_. Therefore, it is probable that SaPIc may only interfere with ɸ2c (Supplementary Fig. S7). In this case, we suggest two possibilities: the SaPI was the last transferred or, more interestingly, the ɸ6c may be the last acquired, since it is not affected by interference mechanisms.

DUF3113, identified as the SaPImw2 derepressor, is a small, non-essential protein and is sufficient for SaPImw2 derepression. All derepressor phage proteins have a dual role: a primary function related to the phage biology and a secondary function related to SaPI mobilisation. The best studied example is the dUTPase protein, which prevents the incorporation of uracil into DNA and is able to mobilise SaPIbov1^25,28,38,40,41^. DUF3113 is a protein with an unknown function for the phage. In our study, deletion of the gene did not change phage replication (data not shown) and did not decrease the phage titre, which indicates that in laboratory conditions, DUF3113 is not an essential protein.

In other phages, DUF3113-homologous proteins were chosen to try to understand the interaction mechanism with the Stl repressor and subsequent SaPI induction. DUF3113_ɸ11_ and DUF3113_ɸ2c_ variants did not derepress SaPImw2. Instead, DUF3113_80α_ and DUF3113_ɸ6c_ variants were more effective at inducing SaPImw2 than DUF3113_ɸSa2mw._ The higher capacity to induce SaPImw2 corresponds with greater affinity for the Stl repressor. Overexpression of DUF3113_80α_ and DUF3113_ɸ6c_ variants in a SaPImw2-positive strain affected bacterial growth because SaPIs overexpression has a high cost for the bacteria. On the other hand, overexpression of DUF3113_ɸSa2mw_ produced normal bacterial growth of the SaPI-positive strains, even though there was SaPImw2 induction. Possibly, the lower capacity to induce the SaPI cycle caused SaPI and the bacteriophage to be in equilibrium within MW2.

As previously mentioned, strain C presents two prophages that seem to be hijacked by SaPIc, which is mobilised in a coordinated way. ɸ6c induces the SaPI, and ɸ2c is involved in packaging. Phage collaboration can also be suspected in the MW2 strain. The SaPI titre from MW2 (ɸSa2mw and ɸSa3mw) was three orders of magnitude higher than that of ɸSa2mw lysogen, which indicates that although ɸSa2mw is responsible for SaPImw2 induction, ɸSa3mw increases SaPImw2 mobilisation. In this case, both phages are *cos* phages, but the *cos* site sequence of ɸSa2mw is identical to that of SaPImw2, so this phage could both induce and encapsidate SaPImw2. However, lysis of the MW2 culture after mitomycin C treatment was higher than that in the ɸSa2mw lysogen. This suggests that ɸSa3mw is only involved in lysis, which improves the mobilisation rate.

In conclusion, our study demonstrates the existence of SaPI mobilisation in clinical *S. aureus* strains. Additionally, the study shows signs of a new SaPIs strategy using the machinery of more than one phage in a coordinated way to increase SaPI transduction. A new derepressor protein has been characterised, but more extensive studies are necessary to identify other derepressor proteins and to better understand the mechanism of SaPI mobilisation and the consequent transfer of virulence factors.

## Methods

### Bacterial growth

The bacterial strains used in this study are listed in Supplementary Table S1*. S. aureus* strains were cultured in tryptic soy broth (TSB) or agar (TSA) plates supplemented with erythromycin (10 μg /ml, Sigma-Aldrich) or chloramphenicol (20 μg /ml, Sigma-Aldrich) as needed*. Escherichia coli* were grown in Luria-Bertani broth (LB) supplemented with ampicillin (100 μg /ml, Sigma-Aldrich) or kanamycin (50 μg /ml, Sigma-Aldrich) as appropriate. Both were grown at 37 °C.

### Phage isolation and transduction

*S. aureus* strains were grown up to OD_540_: 0.2–0.3 and were then treated with mitomycin C (2 μg /ml) to activate the SOS response and induce the resident prophages. Bacterial cultures were incubated at 32 °C and 80 rpm for 4 h or until lysis occurred. The cultures were filtered (0.22 µm filters), and the obtained phage lysates were diluted and spotted on the susceptible prophage-free RN4220 strain^42^. Phage from the centre of an individual plaque were extracted. PCR of the phage integrases was performed to check the lysogeny. GTM752 was obtained using this method.

To test the phage-induced SaPI mobilisation (SaPI o transducing titre), different dilutions of the filtered lysates (100 µl) were used to infect 1 mL of the acceptor strain RN4220 up to OD_540_ = 1.4 at 37 °C for 15 min and plated in TSA containing the corresponding antibiotic. After 24 h at 37 °C, the colonies were counted.

### DNA Methods

DNA manipulations were performed using standard procedures. The primers used in this study are listed in Supplementary Table S3.

Southern blot was performed by standard procedures^43^. For sample preparation, *S. aureus* strains were grown to OD_540_: 0.2–0.3 and were then induced with mitomycin C (2 μg/ml). The cultures were incubated at 32 °C and 80 rpm. Samples were taken 90 minutes after phage induction. Then, 1 ml of the sample was centrifuged, and the pellet was resuspended in 50 µL lysis buffer (47.5 µl of TES-sucrose: 10 mM Tris, 1 mM EDTA, 100 mM NaCl, 0.5 M sucrose and 2.5 µl of lysostaphin (5 mg/ml, Sigma-Aldrich)). After 30 min at 37 °C, 47.5 µl of 2% SDS and 2.5 µl of proteinase K (20 mg/ml) were added, and the mixture was incubated at 55 °C for 30 min. Next, 11 µL of loading buffer dye 10X was added, and the mixture was shaken for 20 min. The samples were cold (N_2_)/heat (65 °C) treated three times.

The standard SDS minilysates were resolved on a 0.7% agarose gel at 20 V overnight, transferred to a nylon membrane (Roche) via capillary action and blotted with specific Digoxigenin-labelled probes and anti-DIG antibodies. Specific primers (Supplementary Table S3) for SaPI integrase genes were used to generate DIG-labelled probes. Detection was carried out according to the Immun Star^TM^ AP substrate pack (BIO-RAD).

For Western blot, the strains were treated with CdCl_2_ (2 μM) and harvested (1 ml) after 2 h. The pellets were resuspended in 200 μl lysis buffer (50 mM Tris-HCl, 20 mM MgCl_2_, 30% raffinose and 1 μl of lysostaphin) and incubated 1 h at 37 °C. SB buffer with β-mercaptoethanol was added, and the samples were heated 10 min at 95 °C. Samples were electrophoresed on a 15% SDS-PAGE gel, transferred to a PVDF membrane and incubated with 5% non-fat milk in TBST (10 mM Tris, pH 8.0, 150 mM NaCl, 0.5% Tween 20) for 60 min. The membrane was washed with TBST, incubated with anti-FLAG antibodies (Sigma-Aldrich) and revealed according to the protocol supplied by the manufacturer.

To delete the DUF3113 gene and introduce the Cm marker in SaPImw2, we used the plasmid pMAD^44^, as previously described^25^. pGTM1 was constructed by 3-piece and pGTM21 by 2-piece overlap assembly PCR. The primer pairs used are listed in Supplementary Table S3. The whole fragments were subsequently cloned into the pMAD vector, and the plasmids were transformed by electroporation into *S. aureus* RN4220 and transduced to MW2 and different lysogens. Allelic replacement was carried out by a two-step procedure. First, the pMAD plasmid was integrated into the chromosome by homologous recombination under non-permissive conditions (44 °C), and in the second step, the plasmid was avoided under a permissive temperature (30 °C). Mutation was confirmed by DNA sequencing.

### Plasmid construction

The plasmids expressing allelic variants of DUF3113 proteins were prepared by cloning PCR products obtained with the oligonucleotide primers listed in Supplementary Table S3. Proteins were expressed in *S. aureus* under inducing conditions from the cadmium-inducible promoter (P*cad*) in the expression vector pCN51, as previously described^25^.

### β-lactamase activity assay

The plasmid pCN41 was used to clone the intergenic regulatory region of SaPImw2 fused to the β-lactamase reporter gene contained in this plasmid, which generated pGTM2. A cadmium-inducible promoter was introduced into the pCU1 plasmid and used to overexpress allelic variants of the DUF3113 protein. Plasmids were electroporated into RN4220 pGTM2, and the transformed strains were grown up to OD_540_: 0.2 and induced with CaCl_2_ (1 µM). Lysogens of different phages were also transformed with pGTM2 and induced with mitomycin C. Samples were taken after 2 h for a β-lactamase activity assay using nitrocefin as a substrate. Changes of the OD at 490 nm were registered during 20 min (every 30 s) in the Synergy H1 microplate reader. β-lactamase activity units were calculated using the formula (ΔVmax(OD_490_)*1000/min)/OD_650._

### Protein expression and purification

Stl and DUF3113 genes were amplified by PCR and cloned into the pET28a vector. The primers were designed to generate an N-terminal 6xHis-tag fusion Stl protein and a 6xHis-tag fusion DUF3113 protein. The plasmids were transformed into *E. coli* BL21(DE3). *E. coli* cells containing the recombinant plasmids were propagated in 500 ml of LB broth (50 µg/ml kanamycin). Cultures were grown until the OD_650_ reached 0.6–0.8, then isopropyl-β-D-thiogalactopyranoside (IPTG, Thermo Scientific) was added to reach a concentration of 1 mM. After induction, the cultures were grown overnight at 16 °C and 200 rpm. Cells were harvested by centrifugation (5000 g for 10 min at 4 °C) and resuspended in phosphate buffer (20 mM Na-phosphate pH 7,4, 300 mM NaCl, 10% glycerol and 0,1 mM PMSF). The cells were lysed by sonication (Amplitude 70% 3 min: 3 s ON and 10 s OFF) and centrifuged (11000 g for 10 min). Supernatants were applied to a metal affinity resin (TALON, Clontech) and incubated for one hour at RT. The hexahistidine-tagged proteins were eluted with 100–500 mM imidazole gradient buffers. Elution fractions were analysed by SDS/PAGE and by size-exclusion chromatography.

For pull-down assays, the primers were designed to generate N-terminal 6xHis-tag fusion Stl proteins and untagged DUF3113 proteins. The 6xHis-tag Stls were incubated for 1 h with metal affinity resin and for 1 h with untagged DUF3113 proteins. Pull-down elution was performed with 250 mM imidazole buffer. Elution fractions were analysed by SDS/PAGE and by size-exclusion chromatography.

### Size-exclusion chromatography

A Superdex^TM^ 75 26/60 column running on a ÄKTA^TM^ pure25 system (GE Healthcare Life Sciences) was used to study the interaction of the selected proteins. The column was equilibrated in 20 mM phosphate buffer containing 300 mM NaCl at pH 7.4, and the elution was performed at 2.6 ml/min at 25 °C. The protein concentration was 17 μM for 6xHis-tag Stl_SaPImw2_ and 40 μM for 6xHis-tag DUF3113_ɸSa2mw_. The injection volume was 7.5 mL. Calibration of the column was performed with aldolase (158 KDa), covalbumina (75 KDa), BSA (66 KDa), ovalbumina (44 KDa), dimer ribonuclease (27.4 KDa), dimer ubiquitin (17 KDa), ribonuclease (13.5 KDa) and ubiquitin (8.5 KDa) (Supplementary Fig S6). Peak protein fractions were analysed by SDS-PAGE.

## Electronic supplementary material

Supplementary Information
