# Supplementary material for: Mobilisation Mechanism of Pathogenicity Islands by Endogenous Phages in Staphylococcus aureus clinical strains
Source: Sci Rep. 2018 Nov 13;8:16742. doi: 10.1038/s41598-018-34918-2 (PMC6233219; doi:10.1038/s41598-018-34918-2)
Supplement: Supplementary file 1 — Supplementary Information [file 41598_2018_34918_MOESM1_ESM.pdf]

Supplementary information

Mobilisation Mechanism of Pathogenicity Islands by Endogenous Phages in *Staphylococcus aureus* clinical strains

Mercedes Cervera-Alamar[1], Katerina Guzmán-Markevitch[1], Miglė Žiemytė[1], Leticia Ortí[2,3], Patricia Bernabé-Quispe, Antonio Pineda-Lucena[2,3], Javier Pemán[1,4] and María Ángeles Tormo-Mas[1*]

[1] Severe Infection Group, Health Research Institute Hospital La Fe, Valencia, Spain

[2]Drug Discovery Unit, Health Research Institute Hospital La Fe, Valencia, Spain.

[3]Joint Research Unit in Clinical Metabolomics, Príncipe Felipe Research Center

/ Health Research Institute Hospital La Fe, Valencia, Spain

[4] Microbiology Department, Polytechnic Universitary Hospital La Fe, Valencia, Spain

[*]Correspondence:

Mª Ángeles Tormo Mas
tormo_man@iislafe.es
+34961246676

Supplementary Figures

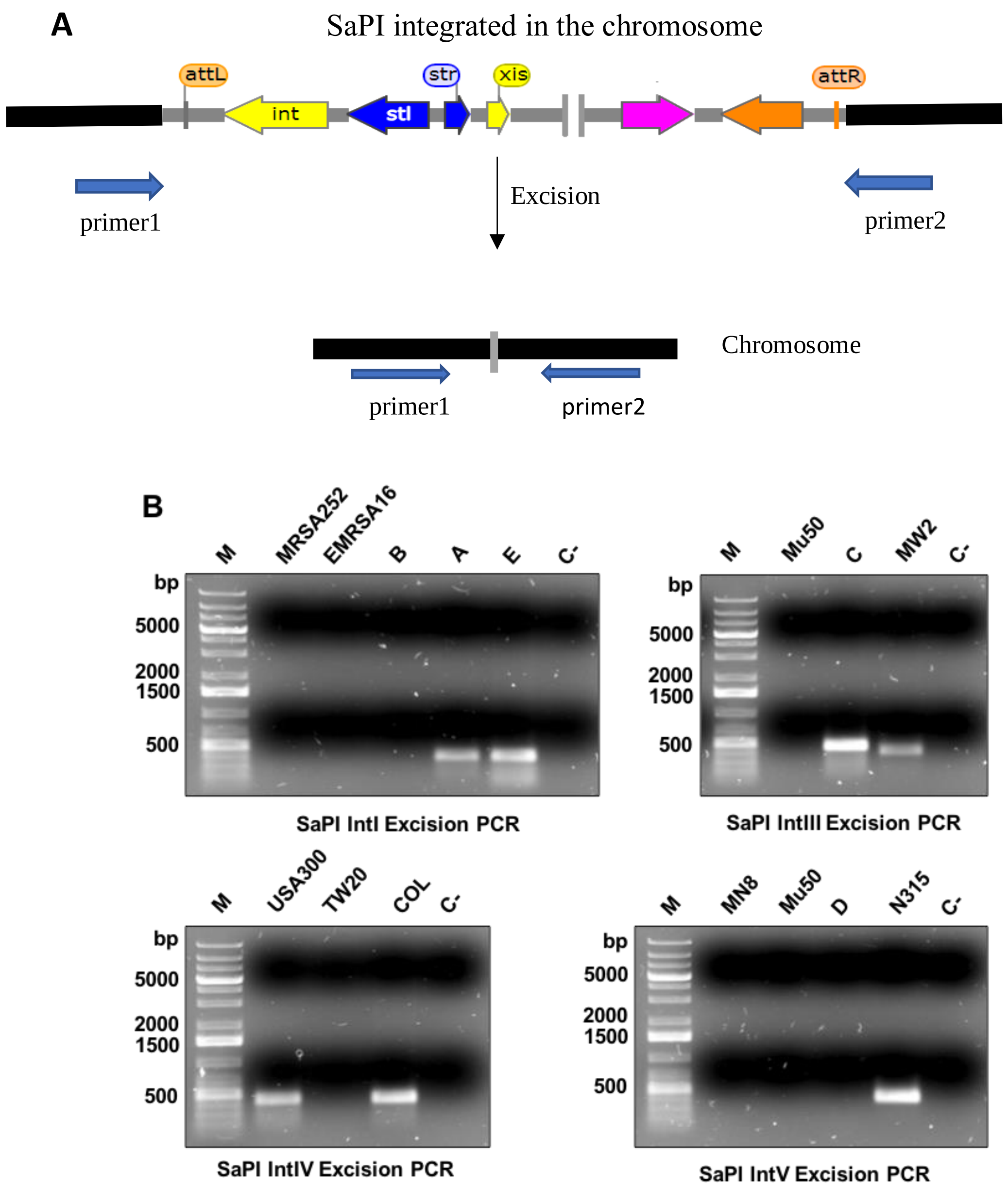

Figure S1. A. Schematic representation of SaPI excision from the chromosome and PCR primers used for the identification. Specific primers recognizing the flanking region of each SaPI type were used. B. Detection of SaPI excision by PCR amplification. Strains A, E, C, MW2, USA300, COL and N315 were positive for excision.

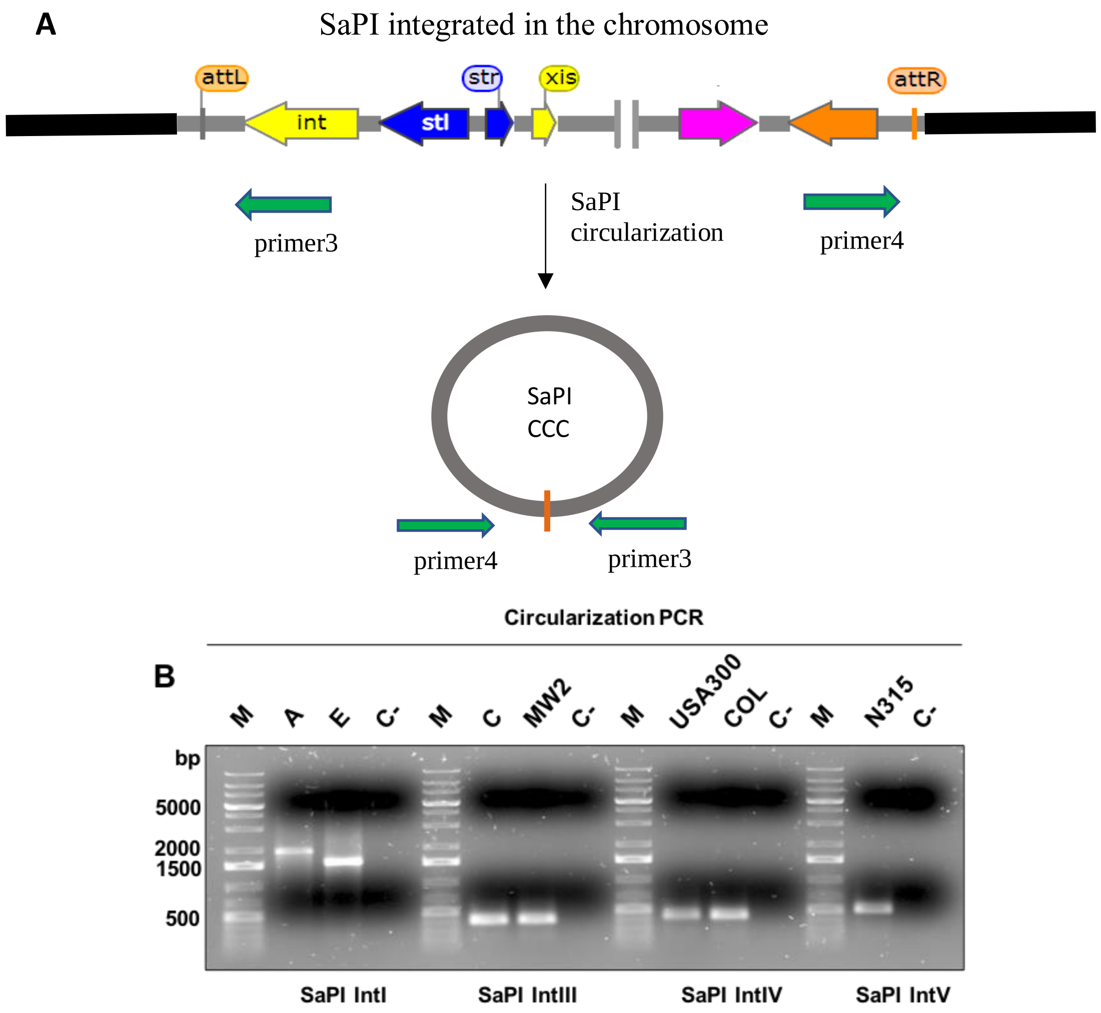

Fig. S2. A. Schematic representation of SaPI circularization and PCR primers used. The primers were designed specifically for each SaPI. B. Detection of SaPI circularization by PCR amplification using a pair of primers set divergently at both termini of SaPI. Only excised SaPIs (Fig. S1) were tested for circularization and all result were positive.

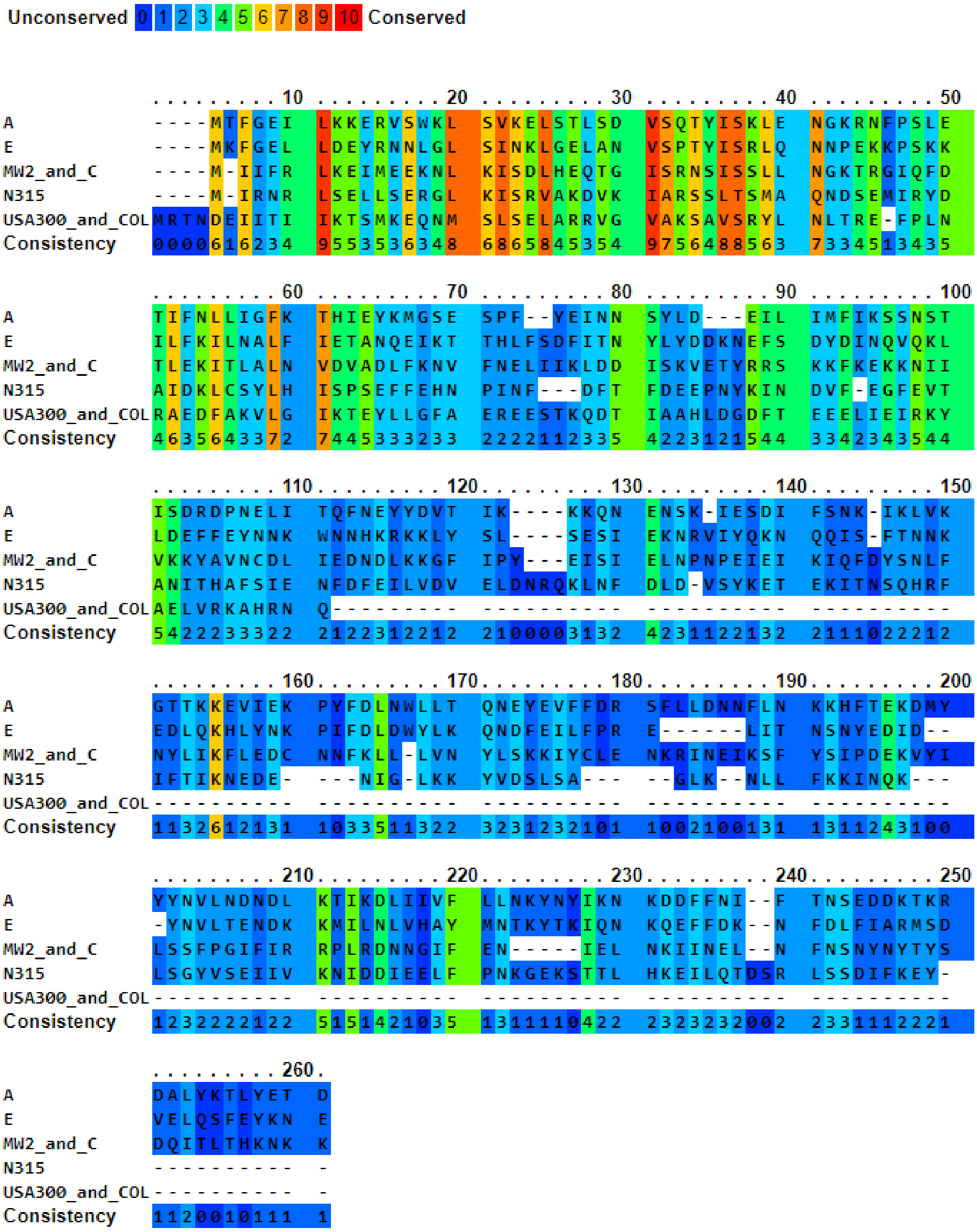

Figure. S3. Stl amino acid sequence from the studied SaPIs were aligned using the PRALINE[1] multiple sequence alignment program.

```
phiII_mw2   -TTTACTTCT CCGTTTTATA TGAAGGATTA G-GAGT-GTG AT--TGAATG CAACATCAAG  55
plaque1     ---CT.G.TC T......... .......... T-....-... ..--..C... ..........  53
plaque2     T.CAGTACG. A...CG.... .......... .C....A..A ..GT..C..C ..........  60

phiII_mw2   CTTATATCAA TGCTTCTGTT GACATTAGAA TTCCTACAGA AGTCGAAAGT GTTAATTACA  115
plaque1     .......... .......... .......... .......... .......... ..........  113
plaque2     .......... .......... .......... .......... .......... ..........  120

phiII_mw2   ATCAGATTGA TAAAGAAAAA GAGAATTTGG CGGACTATTT ATTTAATAAT CCAGGTGAAC  175
plaque1     .......... .......... ..A....... .......... .......... ..........  173
plaque2     .......... .......... ..A....... .......... .......... ..........  180

phiII_mw2   TATTAAAATA TAACGTTATA AATATCAAGG TTTTAGATTT AGAGGTGGAA TGATGG 231
plaque1     .......... .......... .......... .......... .......... ...... 229
plaque2     .......... .......... .......... .......... .T........ ...... 236
```

Figure S4. Sequence alignment of DUF3113 gene and its ribosome binding site from two obtained plaques in comparison with DUF3113$_{\phi Sa2mw}$ wt.

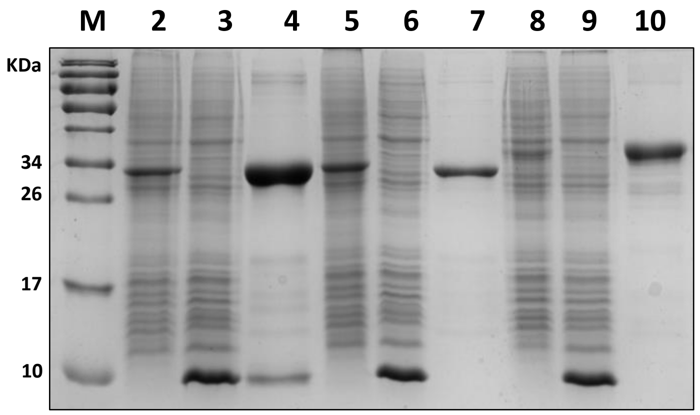

Figure. S5. Affinity chromatography of DUF3113 for His-tagged SaPImw2 Stl. Lane 1: marker. Lane 2 3, 5, 6, 8 and 9: controls of different proteins induction with 1mM of isopropyl-β-d-thiogalactoside (IPTG) after 20 hours at 16°C. Lane 4: pull down of His$_6$-Stl$_{SaPImw2}$ (32.52 kDa) with DUF3113$_{\varphi Sa2mw}$ (7.1 kDa) Lane 7: pull down of His$_6$-Stl$_{SaPImw2}$ (32.52 kDa) with DUF3113$_{\varphi 2c}$. Lane 10: pull down of His$_6$-Stl$_{SaPIbov1}$ (35 kDa) with DUF3113$_{\varphi Sa2mw}$.

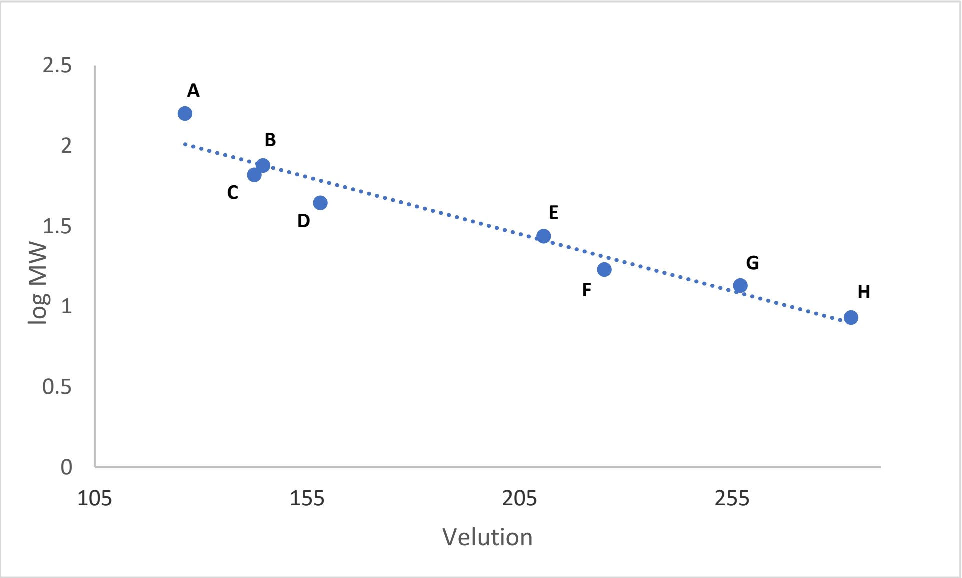

Figure S6. Calibration curve of a Superdex™ 75 26/60 column running on a ÄKTA™ pure25 system (GE Healthcare Life Sciences) ($y = -0{,}0071x + 2{,}9052$; $R^2 = 0{,}9445$). The column was equilibrated with 20mM Phosphate 300mM NaCl pH 7.4, the flow rate 2.6 ml/min; and injection volume 1 ml. Calibration of the column was performed with, (A) aldolase (158 kDa), (B) covalbumina (75 kDa), (C) BSA (66 kDa), (D) ovalbumina (44 kDa), (E) dimer ribonuclease (27.4 kDa), (F) dimer ubiquitin (17 kDa), (G) ribonuclease (13.5 kDa) and (H) ubiquitina (8.5 kDa).

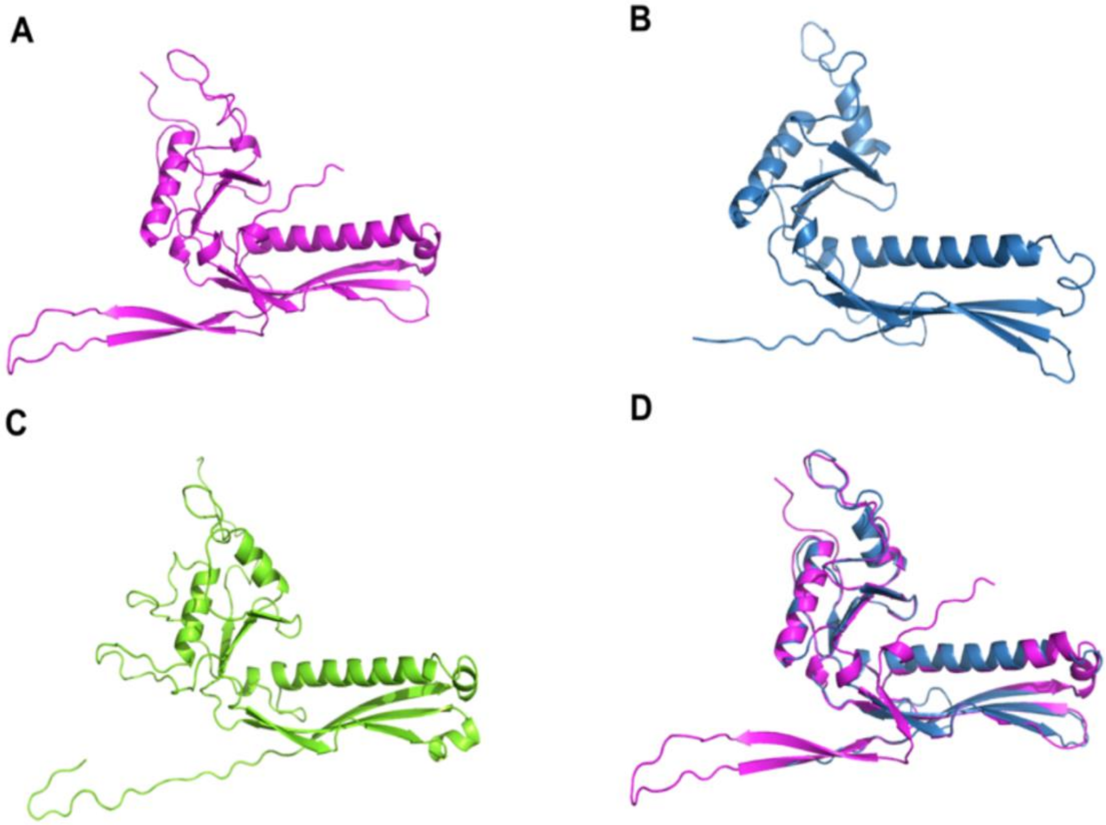

Fig. S7. Cartoon representation of (A) Ccm SaPIc (residues 156-351), (B) CP phi2c (residues 156-401) and (C) CP phi6c (residues 156-401). Structural models for these proteins were generated using RaptorX[2]. Superposition of Ccm SaPIc and CP phi2c (D), using PyMol[3], exhibited a root-mean-square-deviation (RMSD) of 0.511. The same comparison involving Ccm SaPIc and CP phi6c revealed a significant higher value (14.622).

Supplementary Tables

Table S1. Strains used in this study

Strain	Description	Reference
A		[4]
B		[4]
C		[4]
D		[4]
E		[4]
COL		[5]
E-MRSA 16		[6]
MRSA252		[6]
MW2		[7]
Mu50		[8]
MN8		[9]
N315		[8]
TW20		[10]
USA300		[11]
RN4220	Restriction-defective derivate of RN450	Lab strain
DH5α	Recombination-deficient cloning strain	Lab strain
BL21(DE3)	Expression strain	Stratagene
RN10359	RN4220 (80α)	Lab strain
RN451	RN450 (phi11)	Lab strain
GTM750	MW2 SaPImw2 *ent*C*::cat*	This study
GTM751	RN4220 SaPImw2 *ent*C*::cat*	This study
GTM752	RN4220 lysogenic for ϕSa2mw	This study
GTM753	GTM752 SaPImw2 *ent*C*::cat*	This study
GTM754	GTM752 pGTM2	This study
GTM755	RN4220 pGTM2	This study
GTM756	MW2 ΔDUF3113 SaPImw2 *ent*C*::cat*	This study
GTM757	RN4220 φSa2mw ΔDUF3113 SaPImw2 *ent*C*::cat*	This study
GTM758	RN4220 φSa2mw ΔDUF3113	This study
GTM759	GTM757 pGTM20	This study
GTM760	GTM755 pGTM4	This study
GTM761	GTM755 pGTM5	This study
GTM762	GTM755 pGTM6	This study
GTM763	GTM755 pGTM7	This study
GTM764	GTM755 pGTM8	This study
GTM765	GTM755 pGTM9	This study
GTM766	GTM755 pGTM10	This study
GTM767	GTM755 pGTM11	This study
GTM768	GTM755 pGTM12	This study
GTM769	GTM755 pGTM13	This study
GTM770	GTM755 pGTM14	This study
GTM771	GTM755 pGTM15	This study

GTM772	GTM755 pGTM16	This study
GTM773	GTM755 pGTM17	This study
GTM774	GTM755 pGTM18	This study
GTM775	GTM755 pGTM19	This study
GTM776	BL21 pGTM22	This study
GTM777	BL21 pGTM23	This study
GTM778	BL21 pGTM24	This study
GTM779	BL21 pGTM25	This study
GTM780	BL21 pGTM26	This study
GTM781	RN4220 80α pGTM2	This study
GTM782	RN4220phi11 pGTM2	This study
GTM783	RN4220 phi12 pGTM2	This study
GTM784	RN4220 80α SaPImw2 *ent*C*::cat*	This study
GTM785	RN4220 phi11 SaPImw2 *ent*C*::cat*	This study
GTM786	RN4220 phi12 SaPImw2 *ent*C*::cat*	This study
GTM787	RN4220 pGTM27 SaPImw2 *ent*C*::cat*	This study
GTM788	RN4220 pGTM28 SaPImw2 *ent*C*::cat*	This study
GTM789	RN4220 pGTM29 SaPImw2 *ent*C*::cat*	This study
GTM790	RN4220 pGTM30 SaPImw2 *ent*C*::cat*	This study
GTM791	RN4220 pGTM31 SaPImw2 *ent*C*::cat*	This study
GTM792	RN4220 pGTM2 pGTM32	This study
GTM793	RN4220 pGTM2 pGTM33	This study
GTM794	RN4220 pGTM2 pGTM34	This study
GTM795	RN4220 pGTM2 pGTM35	This study
GTM796	RN4220 pGTM2 pGTM36	This study
GTM797	RN4220 pGTM37 SaPImw2 *ent*C*::cat*	This study
GTM798	RN4220 pGTM38 SaPImw2 *ent*C*::cat*	This study
GTM799	RN4220 pGTM39 SaPImw2 *ent*C*::cat*	This study
GTM800	RN4220 pGTM40 SaPImw2 *ent*C*::cat*	This study
GTM801	RN4220 pGTM2 pGTM41	This study
GTM802	RN4220 pGTM2 pGTM42	This study
GTM803	RN4220 pGTM2 pGTM43	This study
GTM804	RN4220 pGTM2 pGTM44	This study
GTM805	RN4220 SaPImw2 *ent*C*::cat* pGTM20	This study
GTM806	RN4220 SaPImw2 *ent*C*::cat* phi11 ΔDUF3113	This study
GTM807	RN4220 SaPImw2 *ent*C*::cat* phi12 ΔDUF3113	This study

Table S2. Plasmids used in this study

Plasmid	Description	Reference
pCN51	expression vector	[12]
pCN41	expression vector	[12]
pCU1	expression vector	[13]
pET28a	expression vector	Novagen
pGTM1	pMAD *ent*C*::cat*	This study
pGTM2	pCN41 *stlstr*$_{SaPImw2}$ (regulatory region SaPImw2)	This study
pGTM3	pCU1 cadmium- inducible promoter P*cad*	This study
pGTM4	pGTM3 MW_RS07680 (MW1434)	This study
pGTM5	pGTM3 region from MW_RS07670 (MW1432) to MW_RS07660 (MW1430)	This study
pGTM6	pGTM3 region from MW_RS07655 (MW1429) to MW_RS07650 (MW1428)	This study
pGTM7	pGTM3 MW_RS07640 (MW1426)	This study
pGTM8	pGTM3 region from MW_RS07630 (MW1424) to MW_RS07600 (MW1416)	This study
pGTM9	pGTM3 region from MW_RS07590 (MW1414) to MW_RS07570 (MW1410)	This study
pGTM10	pGTM3 MW_RS07555 (MW1406)	This study
pGTM11	pCU1 MW1424 (DUF3113)	This study
pGTM12	pGTM3 MW1423	This study
pGTM13	pGTM3 MW1422	This study
pGTM14	pGTM3 MW1421	This study
pGTM15	pGTM3 MW1420	This study
pGTM16	pGTM3 MW1419	This study
pGTM17	pGTM3 MW1418	This study
pGTM18	pGTM3 MW1417	This study
pGTM19	pGTM3 MW1416	This study
pGTM20	pCN51 MW1424 (DUF3113)	This study
pGTM21	pMAD ΔDUF3113 ϕSa2mw	This study
pGTM22	pET28a-Stl$_{SaPImw2}$ (C-terminal His6 tag)	This study
pGTM23	pET28a DUF3113$_{\phi Sa2mw}$ (C-terminal His6 tag)	This study
pGTM24	pET28a DUF3113$_{\phi Sa2mw}$	This study
pGTM25	pET28a DUF3113 $_{\phi Sa2c}$	This study
pGTM26	pET28a-Stl$_{SaPIbov1}$ (C-terminal His6 tag)	This study
pGTM27	pCN51_ DUF3113$_{\phi Sa2mw}$ _3xflag	This study
pGTM28	pCN51_DUF3113$_{80\alpha}$_3xflag	This study
pGTM29	pCN51_DUF3113$_{\phi 11}$_3xflag	This study
pGTM30	pCN51_DUF3113$_{\phi 2c}$_3xflag	This study
pGTM31	pCN51_ DUF3113$_{\phi 6c}$ _3xflag	This study
pGTM32	pGTM3_ DUF3113$_{\phi Sa2mw}$ _3xflag	This study
pGTM33	pGTM3_ DUF3113$_{80\alpha}$ _3xflag	This study
pGTM34	pGTM3_ DUF3113$_{\phi 11}$ _3xflag	This study
pGTM35	pGTM3_ DUF3113$_{\phi 2c}$ _3xflag	This study

pGTM36	pGTM3_ DUF3113$_{\phi 6c}$ _3xflag	This study
pGTM37	pCN51_RBS_ DUF3113$_{80\alpha}$ _3xflag	This study
pGTM38	pCN51_RBS_ DUF3113$_{\phi 11}$ _3xflag	This study
pGTM39	pCN51_RBS_ DUF3113$_{\phi 2c}$ _3xflag	This study
pGTM40	pCN51_RBS_ DUF3113$_{\phi 6c}$ _3xflag	This study
pGTM41	pGTM3_RBS_ DUF3113$_{80\alpha}$ _3xflag	This study
pGTM42	pGTM3_RBS_ DUF3113$_{\phi 11}$ _3xflag	This study
pGTM43	pGTM3_RBS_ DUF3113$_{\phi 2c}$ _3xflag	This study
pGTM44	pGTM3_RBS_ DUF3113$_{\phi 6c}$ _3xflag	This study

Table S3. Primers used in this study. Restriction sequences are underlined.

Plasmid	oligonucleotides	Sequence
pGTM1 (pMAD *ent*C::*cat*)	SaPImw2_1mB	CGCGGATCCGTTTCAGCAGCTTTTACAACG
	SaPImw2_2c	CTTTCTTATCTTGATAATAAGGAGAAACAGAG GATTTCTAAGC
	SaPImw2_3m	CCCCGTTAGTTGAAGAAGGGGATAATGTTAAT CCGATTTTG
	SaPImw2_4cE	CCGGAATTCAGTCTTATCTAACGGCGATG
	cat194_1m	CTTATTATCAAGATAAGAAAG
	cat194_2c	CCTTCTTCAACTAACGGGGG
pGTM21 (pMADΔDUF3113)	duf3113_mw2_5mB	CGCGGATCCAGATGTGTTCAACACACACGG
	duf3113_mw2_6c	CATTCAATCACACTCCTAATCC
	duf3113_mw2_7m	GGATTAGGAGTGTGATTGAATGGGTGAACTAT TAAAATATAACG
	phiII_mw2_21cE	CCGGAATTCGTTGCCATCGTTACCCCTC
pGTM2	SaPImw2_5mS	ACGCGTCGACTAATTCGTCCATCATTTCGTTG
	SaPImw2_6cB	CGCGGATCCATGCCGTACACACTAAAAGC
pGTM4	phiII_mw2_1mB	CGCGGATCCTTACAGAGTTTCCAAAATGTC
	phiII_mw2_2cE	CCGGAATTCTGTTGTTTACTTTGAAAATGAG
pGTM5	phiII_mw2_3mB	CGCGGATCCTCATCAGATATCAAGGGCATG
	phiII_mw2_4cE	CCGGAATTCTTGCACTGTTACTTGCTCC
pGTM6	phiII_mw2_5mB	CGCGGATCCAAGTAACAGTGCAAGATGAG
	phiII_mw2_6cE	CCGGAATTCTGCATGAGCTCTATTTGAGTG
pGTM7	phiII_mw2_7mB	CGCGGATCCTTAACACTTGCTACCGAGTCG
	phiII_mw2_8cE	CCGGAATTCAATTAAAGTGGGGCTAAAACC
pGTM8	phiII_mw2_9mS	ACGCGTCGACGATTGAATTTGAATAGTGACGG
	phiII_mw2_10cB	CGCGGATCCAAGTTCTTTTAATTGATCTACTG
pGTM9	phiII_mw2_11mS	ACGCGTCGACGACGCAGGAAAAGGATACGTG
	phiII_mw2_12cB	CGCGGATCCAATCCTGGAGAGGGCTTTAGC
pGTM10	phiII_mw2_13mB	CGCGGATCCTAGTGTTAGGGAGTAAAGAGG
	phiII_mw2_14cB	CCGGAATTCGATAGCTATGTGGTTTGAAGTC
pGTM11/pGTM20	phiII_mw2_9mS	ACGCGTCGACGATTGAATTTGAATAGTGACGG
	phiII_mw2_17cB	CGCGGATCCTCTTCTAGCCATCATTCCACC
pGTM12	phiII_mw2_18mB	CGCGGATCCAGGTGAACTATTAAAATATAAC
	phiII_mw2_19cE	CCGGAATTCATGCTCATTATGCTTCACTCC
pGTM13	phiII_mw2_20mB	CGCGGATCCTCCGTACTGGTTCGATGTCAC
	phiII_mw2_21cE	CCGGAATTCGTTGCCATCGTTACCCCTC
pGTM14	phiII_mw2_22mB	CGCGGATCCATGAGGATTTAGCAAAGGCG
	phiII_mw2_23cE	CCGGAATTCCTTTTAACAATTAGGCAGTCC
pGTM15	phiII_mw2_24mB	CGCGGATCCAGACAAGCATAAGGAATGAAG
	phiII_mw2_25cE	CCGGAATTCCTCTAAAATAAAGTTAGTTGC
pGTM16	phiII_mw2_26mB	CGCGGATCCAGCAACATGGTTTGGAATACG
	phiII_mw2_27cE	CCGGAATTCACTAATACTCATTTTCCTGC
pGTM17	phiII_mw2_28mB	CGCGGATCCAGGTATTGAACTTGATGAAGC
	phiII_mw2_29cE	CCGGAATTCTTCCATTCCACTCACTCGTCC

pGTM18	phiII_mw2_30mB	CGCGGATCCTGTCTGATGATTCAGTTATTAG
	phiII_mw2_31cE	CCGGAATTCAAGCATTTACTCGTCCTCC
pGTM19	phiII_mw2_32mS	ACGCGTCGACTTGCTAACGAGCATGAGATTG
	phiII_mw2_10cB	CGCGGATCCAAGTTCTTTTAATTGATCTACTG
	IntI_F	GATAGCAAAGTACTCATCATCAG
	IntI_R	CATTGTCTGTCTTGATTG
	IntII_F	GACTGCCTATATGTTTG
	IntII_R	CGTTGATATGTGCTTTCTC
	IntIII_F	CTCGTAAGTACATAACAC
	IntIII_R	CTTCATTGCATGATTCAG
	IntIV_F	AGGATTCAAAATGGAATGATGG
	IntIV_R	CCTTATCCATCTGTTCAGTAACG
	IntV_F	GTCTGCTATAGACTTTGAG
	IntV_R	CAATGAGTATGACGAAATG
	SaPI_Int1exc_1m	GCAGGTGGACCAAGAAGAGG
	SaPI_Int1exc_2c	CTTTGTGCAAATCCCAAACAC
	SaPI_Int1circ_1c	ATCTACACTTGCGCAATTAGG
	SaPI_Int1circ_2m	AGGCGTAATTGTGAAACTAGG
	SaPI_Int3exc_1m	TGCTGTTAACGCGATTCAAC
	SaPI_Int3exc_2c	CTTTTTTGACACTTATTTGACAC
	SaPI_Int3circ_1c	ATGACACCAGCTTTTTGGGT
	SaPI_Int3circ_2m	GAGGTGATGTTGGGTGCAC
	SaPI_Int4exc_1m	TCGAACTTTGCAATCGAACA
	SaPI_Int4exc_2c	CTTGAGGGAGTGGGACTGAA
	SaPI_Int4circ_1c	ATGGATCGTGTAGGCCATTC
	SaPI_Int4circ_2m	GTGTGGTTGTTTTCAAGGGC
	SaPI_Int5exc_1m	GCTGCTACAAACGAGTGGGT
	SaPI_Int5exc_2c	ATACCATAACTCCCGCACCA
	SaPI_Int5circ_1c	TACAAGAACGCTTGCGACAC
	SaPI_Int5circ_2m	TATGAGGTGATGTTGGGTGC
pET28a-$Stl_{SaPImw2}$ (C-terminal His6 tag) pGTM22	Stl_SaPImw2_1B	CGCGGATCCATGATAATTTTTCGATTAAAAGAAAT
	Stl_SaPImw2_2cE	CCGGAATTCGGCAATCACCTATTTCATTTC
pET28a_ $DUF3113_{\phi Sa2mw}$ pGTM24	duf3113_mw2_3mS	ACGCGTCGACTATAATAATAATTTGTTTAACTTTAAGAAGGAGATATACCATGCAACATCAAGCTTATATC
	duf3113_4cN	ATAAGAATGCGGCCGCTCTTCTAGCCATCATTCCACC
pET28a_ $DUF3113_{\phi Sa2mw}$ (C-terminal His6 tag) pGTM23	duf3113_8mB	CGCGGATCCATGCAACATCAAGCTTATATC
	duf3113_9cS	ACGCGTCGACTTATAACTTTTCTTCTAGCC

pET28_ DUF3113$_{\phi Sa2c}$ pGTM25	duf3113_phi11_1mS	ACGCGTCGACTATAATAATAATTTGTTTAACTTTAAGAAGGAGATATACCATGCAACAACAAGCATATATAAACG
	duf3113_phi11_2cN	ATAAGAATGCGGCCGCGTTCAACTTCATTTATTCCAC
pET28_ Stl$_{SaPIbov1}$ (C-terminal His6 tag) pGTM26	Stl_SB1_1mB	CGCGGATCCATCGAAGGAGCTGGTCAAATG
	Stl_SB1_2cE	CCGGAATTCTACCTTGTGGCGTGATGAT
pCN51/pGTM3_duf3113 $_{\phi Sa2mw}$ _3xflagC pGTM27 pGTM32	phiII_mw2_9mS	ACGCGTCGACGATTGAATTTGAATAGTGACGG
	phiII_mw2_3xflagC_1cB	CGCGGATCCTTATTTATCATCATCATCTTTATAATCGATATCGTGATCTTTATAATCATCGTGATCTTTATAATTTCCACCTCTAAATCTAAAACC
pCN51/pGTM3_ duf3113φ11 3xflagC pGTM28 pGTM33	phiV_FQ166_37mS	AGCCGTCGACAAGAATGGATGGTATATGAGG
	phi11_3xflagC_1cB	CGCGGATCCTTATTTATCATCATCATCTTTATAATCGATATCGTGATCTTATAATCGCCATGCTGATCTTTATAATCTTCCACCTCTATATATGCATG
pCN51/pGTM3_ duf311380α 3xflagC pGTM29 pGTM34	duf3113_phi80α_1mS	ACGCGTCGACTAGCTCAAAGCGTTATGG
	phi80α_3xflagC_1cB	CGCGGATCCTTATTTATCATCATCATCATCTTTATAATCGATATCGTGATCTTTATAATCGCCATCGTGATCTTTATAATCTTCCACCTCTACATTTACATTT
pCN51/pGTM3_ duf3113φ2.C 3xflagC pGTM30 pGTM35	phiII_mw2_9mS	ACGCGTCGACGATTGAATTTGAATAGTGACGG
	phiII_mw2_3xflagC_1cB	CGCGGATCCTTATTTATCATCATCATCTTTATAATCGATATCGTGATCTTTATAATCATCGTGATCTTTATAATTTCCACCTCTAAATCTAAAACC
pCN51/pGTM3_ duf3113φ6.C 3xflagC pGTM31 pGTM36	duf3113_phi80α_1mS	ACGCGTCGACTAGCTCAAAGCGTTATGG
	duf3113_3xflagC_phiVI_1cB	CGCGGATCCTTATTTATCATCATCATCTTTATAATCGATATCGTGATCTTTATAATCGCCATCGTGATCTTTATAATTTTTCACCTCTACATTTACGTTT
pCN51/pGTM3_ duf3113φ11 3xflagC pGTM38 pGTM42	duf3113_phi11_5m	GAAGGATTAGGAGTGTGATTGAATGCAACAACAAGCATATATAAACG
	phi11_3xflagC_1cB	CGCGGATCCTTATTTATCATCATCATCTTTATAATCGATATCGTGATCTTATAATCGCCATGCTGATCTTTATAATCTTCCACCTCTATATATGCATG
	duf3113_mw2_13mS	ACGCGTCGACGATTGAATTTGAATAGTGACGGATTTACTTCTCCGTTTTATATGAAGGATTAGGAGTGTGATTGAATGC
	phi11_3xflagC_1cB	CGCGGATCCTTATTTATCATCATCATCTTTATAATCGATATCGTGATCTTATAATCGCCATGCTGATCTTTATAATCTTCCACCTCTATATATGCATG
pCN51/pGTM3_ duf311380α 3xflagC pGTM37 pGTM41	duf3113_80α_3m	GAAGGATTAGGAGTGTGATTGAATGCAACAGCAAGCATATATAAACG
	phi80α_3xflagC_1cB	CGCGGATCCTTATTTATCATCATCATCTTTATAATCGATATCGTGATCTTTATAATCCCCATCGTGGATCTTTATAATCTTCCACCTCTACATTTACATTT
	duf3113_mw2_13mS	ACGCGTCGACGATTGAATTTGAATAGTGACGGATTTACTTCTCCGTTTTATATGAAGGATTAGGAGTGTGATTGAATGC
	phi80α_3xflagC_1cB	CGCGGATCCTTATTTATCATCATCATCTTTATAATCGATATCGTGATCTTTATAATCCCCATCGTGGATCTTTATAATCTTCCACCTCTACATTTACATTT
	duf3113_phiII_cepaC_2m	GAAGGATTAGGAGTGTGATTGAATGCAACATCAAGCTTATATC

pCN51/pGTM3_duf3113φ2.C 3xflagC pGTM39 pGTM43	phiII_mw2_3xflagC_1cB	CGCGGATCCTTATTTATCATCATCATCTTTATAATCGATATCGTGATCTTTATAATCATCGTGATCTTTATAATTTCCACCTCTAAATCTAAAACC
	duf3113_mw2_13mS	ACGCGTCGACGATTGAATTTGAATAGTGACGGATTTACTTCTCCGTTTTATATGAAGGATTAGGAGTGTGATTGAATGC
	phiII_mw2_3xflagC_1cB	CGCGGATCCTTATTTATCATCATCATCTTTATAATCGATATCGTGATCTTTATAATCATCGTGATCTTTATAATTTCCACCTCTAAATCTAAAACC
pCN51/pGTM3_duf3113φ6.C 3xflagC pGTM40 pGTM44	duf3113_phi11_5m	GAAGGATTAGGAGTGTGATTGAATGCAACAACAAGCATATATAAACG
	duf3113_3xflagC_phiVI_1cB	CGCGGATCCTTATTTATCATCATCATCTTTATAATCGATATCGTGATCTTTATAATCGCCATCGTGATCTTTATAATTTTTCACCTCTACATTTACGTTT
	duf3113_mw2_13mS	ACGCGTCGACGATTGAATTTGAATAGTGACGGATTTACTTCTCCGTTTTATATGAAGGATTAGGAGTGTGATTGAATGC
	duf3113_3xflagC_phiVI_1cB	CGCGGATCCTTATTTATCATCATCATCTTTATAATCGATATCGTGATCTTTATAATCGCCATCGTGATCTTTATAATTTTTCACCTCTACATTTACGTTT

References

1. Simossis, V. A. & Heringa, J. PRALINE: A multiple sequence alignment toolbox that integrates homology-extended and secondary structure information. *Nucleic Acids Res.* **33,** (2005).

2. Källberg, M. *et al.* Template-based protein structure modeling using the RaptorX web server. *Nat. Protoc.* **7,** 1511–1522 (2012).

3. DeLano, W. L. The PyMOL Molecular Graphics System. *Schrödinger LLC wwwpymolorg* **Version 1.,** http://www.pymol.org (2002).

4. Viana, D. *et al.* A single natural nucleotide mutation alters bacterial pathogen host tropism. *Nat. Genet.* **47,** 361–6 (2015).

5. Gill, S. R. *et al.* Insights on Evolution of Virulence and Resistance from the Complete Genome Analysis of an Early Methicillin-Resistant Staphylococcus aureus Strain and a Biofilm-Producing Methicillin-Resistant Staphylococcus epidermidis Strain. *J. Bacteriol.* **187,** 2426–2438 (2005).

6. Holden, M. T. G. *et al.* Complete genomes of two clinical Staphylococcus aureus strains: Evidence for the rapid evolution of virulence and drug resistance. *Proc. Natl. Acad. Sci.* **101,** 9786–9791 (2004).

7. Baba, T. *et al.* Genome and virulence determinants of high virulence community-acquired MRSA. *Lancet* **359,** 1819–1827 (2002).

8. Kuroda, M. *et al.* Whole genome sequencing of meticillin-resistant Staphylococcus aureus. *Lancet (London, England)* **357,** 1225–40 (2001).

9. Schlievert, P. M. & Blomster, D. A. Production of staphylococcal pyrogenic exotoxin type C: Influence of physical and chemical factors. *J. Infect. Dis.* **147,** 236–242 (1983).

10. Holden, M. T. G. *et al.* Genome sequence of a recently emerged, highly transmissible, multi-antibiotic- and antiseptic-resistant variant of methicillin-resistant Staphylococcus aureus, sequence type 239 (TW). *J. Bacteriol.* **192,** 888–892 (2010).

11. Diep, B. A. *et al.* Complete genome sequence of USA300, an epidemic clone of community-acquired meticillin-resistant Staphylococcus aureus. *Lancet* **367,** 731–739 (2006).

12. Charpentier, E. *et al.* Novel cassette-based shuttle vector system for gram-positive bacteria. *Appl. Environ. Microbiol.* **70,** 6076–6085 (2004).

13. Konarska-Kozlowska,M. and Iyer,V.N. (1981) Physical and genetic organization of the IncN-group plasmid pCU1. Gene, 14, 195–204.
